# Magnesium bioavailability from mineral waters with different mineralization levels in comparison to bread and a supplement

**DOI:** 10.1080/16546628.2017.1384686

**Published:** 2017-10-04

**Authors:** Inga Schneider, Theresa Greupner, Andreas Hahn

**Affiliations:** ^a^ Institute of Food Science and Human Nutrition, Leibniz University Hannover, Hannover, Germany

**Keywords:** Mineral water, magnesium, bioavailability, mineralization, general nutrition

## Abstract

The aim of the present study was to compare the magnesium bioavailability from four mineral waters with different types of mineralization (e.g. SO_4_^2-^, HCO_3_^−^, calcium) with the magnesium bioavailability from bread and from a magnesium supplement. A single-center, randomized, controlled trial with a crossover design with 22 healthy men and women was conducted at the Institute of Food Science and Human Nutrition, Leibniz University Hannover, Germany. The participants consumed the six test products providing 100 mg of magnesium each on six examination days with a one-week washout phase in between. The primary outcome variables were the 24 h urinary magnesium excretion, the 24 h urinary magnesium/creatinine ratio, and the area under the curve of serum magnesium levels for 10 h (AUC_0-10h_). No significant differences among groups were observed for either 24 h urinary magnesium excretion or 24 h urinary magnesium/creatinine ratio. Likewise, statistical group comparisons of AUC_0-10h_ for serum magnesium levels revealed no significant differences among the treatment groups. Accordingly, given equivalent magnesium availability from all test products, neither SO_4_^2-^ content nor the content of HCO_3_^−^ or of calcium influenced the bioavailability of magnesium. Thus, mineral water with higher concentrations of magnesium constitutes a calorie-free magnesium source that contributes to optimal magnesium supply.

## Introduction

Magnesium (Mg) is the second most abundant intracellular cation after potassium and the fourth most abundant cation in the human body. This essential mineral is required for a broad range of physiological and biochemical functions. As a cofactor in more than 300 enzymatic reactions, many of which are dependent on ATP, Mg is involved in many key biochemical pathways, including pathways related to macronutrient degradation, oxidative phosphorylation, DNA and protein synthesis, neuromuscular excitability, and the regulation of parathyroid hormone secretion [1–4].

Furthermore, a low Mg intake may increase the risk of depression [5]. Moreover, children with diabetes mellitus have significantly lower Mg levels than in healthy controls [6]. Mg homeostasis depends on Mg intake but is mostly determined by the renal excretion of Mg [7]. Current recommendations for Mg for adults vary from 400–420 mg/d for men and 310–320 mg/d for women in the US (recommended daily allowance, RDA) to 350 mg/d for men and 300 mg/d for women in the EU (adequate intake, AI), and 300 mg/d for men and 270 mg/d for women in the UK (reference nutrient intake, RNI) [8–11]. When usual Mg consumption patterns were analyzed, most population groups consumed less than the RDA. Data from the National Health and Nutrition Examination Survey (NHANES) indicate that in the US 48% of adult males and 46% of adult females do not meet the current dietary recommendation for Mg [12]. However, in Germany Mg is the most supplemented mineral with 59.4%, followed by calcium with 37.0% [13].

Nuts, green leafy vegetables, and whole grains are relatively rich sources of dietary Mg. Nevertheless, these foods only contribute a maximum of approximately 10% of the recommended intake of Mg [14]. According to the second German National Nutrition Survey (NVS II), men and women consume the largest proportion of magnesium over bread after alcohol-free beverages [15]. Certainly, wholemeal bread contains considerable amounts of phytic acid, which lowers the bioavailability of minerals, including iron, zinc, calcium, and Mg [16]. Given the low content of Mg in most foods and the phytic acid content of wholemeal bread, alternative sources of Mg should be preferred. Mineral water (MW) is a promising candidate because it is calorie-free, contains no potential allergens, and ensures hydration. Furthermore, although the Mg content of mineral waters varies widely depending on the water source, it can reach more than 150 mg/l [17].

According to the literature, Mg from mineral water and supplements is bioavailable [17–22]. However, it is unknown whether Mg bioavailability from mineral water is comparable to Mg bioavailability from bread. Furthermore, Mg bioavailability from mineral water may be influenced by concentrations of other minerals, such as calcium, in the water [23,24]. Therefore, the aim of the present study was to compare the Mg bioavailability from four mineral waters with different types of mineralization with Mg bioavailability from bread and from a Mg supplement.

## Materials and methods

### Study design

A single-center, randomized, controlled trial with a crossover design was conducted by trained professionals using standardized methods at the Institute of Food Science and Human Nutrition, Leibniz University Hannover, Germany. The study involved a screening phase and six examinations with a one-week washout phase prior to each examination.

Ethical approval was provided by the Ethics Commission of the Medical Chamber of Lower Saxony (Hannover, Germany). In accordance with the guidelines of the Declaration of Helsinki, written informed consent was obtained from all subjects prior to their participation in the study. This study is registered in the German Clinical Trial Register (DRKS00010411).

### Subjects

Healthy participants were recruited via advertisements from the general population in Hannover, Germany. They were selected according to inclusion and exclusion criteria, which were assessed using questionnaires. The main inclusion criteria were an age between 18 and 50 years and a body mass index (BMI) between 18.5 and 29.9 kg/m^2^. The exclusion criteria were an allergy to any of the test products, the intake of Mg supplements, the regular intake of laxatives, and chronic gastrointestinal disorders or prior gastrointestinal surgical procedures.

### Test products and procedure

Four different mineral waters (MW 1, MW 2, MW 3, and MW 4), whole rye bread, and a supplement containing magnesium carbonate (MgCO_3_) were investigated in this bioavailability study. The mineral concentrations of the six test products are indicated in Table 1. Procedure has already been described in Greupner et al. [25]. In brief, test products were adjusted to provide 100 mg of Mg, with the exception of the Mg supplement, which contained 110 mg of Mg per tablet. As needed, product volumes were reached via the addition of demineralized water to ensure the consumption of equal quantities of fluids across all groups. All participants received each test product in an individually randomized order generated in a Williams design.

**Table 1.** Mineral composition of the six test products (MW 1: mineral water 1; MW 2: mineral water 2; MW 3: mineral water 3; MW 4: mineral water 4; Suppl.: magnesium supplement) .

Mineral value	MW 1	MW 2	MW 3	MW 4	Bread ^a^	Suppl.^b^
Mg^2+^	241 mg/l	108 mg/l	124 mg/l	137 mg/l	531 mg/kg	110 mg/pill
Ca^2+^	168 mg/l	348 mg/l	528 mg/l	290 mg/l	n.a.	-
Na^+^	261 mg/l	118 mg/l	28.8 mg/l	100 mg/l	n.a.	-
Cl^−^	14 mg/l	40 mg/l	28.9 mg/l	181 mg/l	n.a.	-
SO_4_^2-^	17 mg/l	38 mg/l	1463 mg/l	8.8 mg/l	n.a.	-
HCO_3_^−^	2451 mg/l	1816 mg/l	403 mg/l	1519 mg/l	n.a.	-

n.a.: not analyzed; ^a^ whole rye; ^b^ magnesium carbonate (MgCO_3_)

Participants were instructed to minimize their Mg intake two days before each examination and to avoid excessive exercise on the day prior to the examination. A list of restricted foods was given to each participant prior to the intervention.

On the examination days, each participant consumed one of the test products in a randomized order after an overnight fast. Participants were instructed to drink Mg-poor water (8 mg of Mg per liter) at defined time points during the 12 h preceding the first draw of fasting blood. Test products had to be consumed within 30 minutes and were given with a standardized breakfast (9.8 mg Mg/portion). Blood samples were drawn initially and at 1, 2, 3, 4, 5, 6, 8, and 10 h after the intake of the test product. Urine samples were collected pre-dose and at defined intervals up to 24 h after dosing (0–2, 2–4, 4–6, 6–8, 8–12, and 12–24 h). During the experimental period (24 h), the participants consumed standardized Mg-poor meals and water. The total Mg intake from the background diet was 195.2 mg. Additionally, the participants consumed 100 mg from the test products (MW 1, MW 2, MW 3, MW 4, and bread) or 110 mg from the Mg supplement. After the 10 h blood draw, the participants were allowed to drink Mg-poor water at any time, in quantities not exceeding 1 liter.

The primary outcome variables were the 24 h urinary Mg excretion, the 24 h urinary Mg/creatinine excretion ratio, and area under the curve for serum Mg levels for 10 h (AUC_0-10h_). Urinary excretion of Mg and serum concentrations of Mg were examined as secondary outcome variables. The blood and the urine samples were prepared and analyzed by the Hannover Medical Care Center of the LADR network. The blood sample quantities were 10.2 ml of the fasting sample and 2.4 ml of each following sample, which results in a total blood volume of 29.4 ml per day. The urine sample quantities were 1 ml of the fasting sample and 10 ml of the 24 h urine sample. The detection limits and coefficients of variation (CV) for the measurements were 0.02 mmol/l (2.73CV%) for urinary Mg, 0.01 mmol/l (1.15CV%) for serum Mg, and 0.1 µmol/l (1.31CV%) for urinary creatinine.

### Data analysis and statistical methods

Data are presented as the means ± standard deviation (SD) for continuous variables. All serum levels were corrected using their respective baseline levels. AUC_0-10h_ for serum Mg levels were calculated geometrically using the trapezoidal rule, ignoring the area below the baseline. If the Kolmogorov–Smirnov test indicated that the study data were not normally distributed, log transformation was applied, and parametric tests were used. Differences among urinary Mg excretion, urinary Mg/creatinine ratio, and AUC_0-10h_ for serum Mg levels were analyzed using ANOVA for repeated measurements. Mauchly’s test was used to determine sphericity. When sphericity could not be assumed, the Greenhouse–Geisser correction was applied. Values of p ≤ 0.05 were regarded as statistically significant. All statistical analyses were performed using SPSS software (version 23.0; SPSS Inc., Chicago, IL, USA).

## Results

### Study population

Twenty-two healthy males and females (men: n = 11, women: n = 11) participated in the study. The subjects’ mean age was 24.2 ± 3.2 years and their mean BMI was 23.6 ± 2.4 kg/m^2^.

### Serum and urinary concentrations of magnesium and creatinine

**Figure 1.**
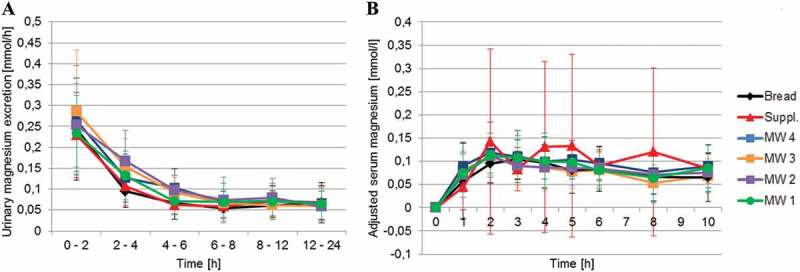
Mean ± SD urinary magnesium excretion (A; MW 1: n = 22, MW 2: n = 20, MW 3: n = 22, MW 4: n = 21, Bread: n = 21, Suppl.: n = 22) and mean ± SD serum magnesium concentrations corrected to baseline (B; MW 1: n = 22, MW 2: n = 21, MW 3: n = 22, MW 4: n = 21, Bread: n = 22, Suppl.: n = 22) after consumption of the test products.

No significant differences among groups were observed for either 24 h urinary Mg excretion or 24 h urinary Mg/creatinine ratio. Likewise, statistical group comparisons of AUC_0-10h_ for serum Mg levels revealed no significant differences among the treatment groups (Table 2). Urinary Mg excretion was initially high and continuously decreased over the following 2 h in all groups. In nearly all groups, after an initial increase, serum Mg levels remained nearly constant after 2 h. Serum Mg levels also increased after consumption of the supplement but fluctuated after 2 h, with no decreases observed (Figure 1).

**Table 2.** Twenty-four hours of urinary magnesium excretion, 24 h urinary magnesium/creatinine ratio, and area under the curve (AUC0-10h) for serum magnesium levels for 10 h (mean ± SD).

	24 h urinary Mg excretion [mmol]		24 h urinary Mg/creatinine ratio [mmol]		AUC_0-10h_ serum Mg	
MW 1	4.41 ± 1.50	n = 22	0.55 ± 0.79	n = 18	0.80 ± 0.35	n = 22
MW 2	4.47 ± 1.17	n = 20	0.43 ± 0.15	n = 18	0.89 ± 0.40	n = 21
MW 3	4.41 ± 1.58	n = 22	0.40 ± 0.08	n = 18	0.78 ± 0.37	n = 22
MW 4	4.68 ± 1.44	n = 21	0.71 ± 1.36	n = 18	0.99 ± 0.39	n = 21
Bread	4.12 ± 1.74	n = 22	0.34 ± 0.07	n = 18	0.81 ± 0.31	n = 22
Suppl.	4.21 ± 1.53	n = 21	0.35 ± 0.07	n = 18	0.82 ± 0.32	n = 22
p	0.194^2^		0.382^a^		0.401^a^	

^a^ sphericity given

^b^ Greenhouse–Geisser correction

## Discussion

The present crossover study, sought to compare the Mg bioavailability from four mineral waters with different types of mineralization with the Mg bioavailability from a Mg supplement, and (assumed for the first time) from bread. In general, Mg was bioavailable from all test products. This finding is consistent with the results of prior studies that revealed Mg bioavailability from mineral water and dietary supplements [17–20,26]. Urinary Mg excretion increased after the consumption of all test products, and 24 h urinary Mg excretion did not significantly differ among the test products. This result is consistent with findings from previous studies that reveal comparable urinary Mg excretion after the intake of mineral water, standard meals, and dietary supplements [17,22,27]. In addition, no significant differences among the test products were found with respect to the Mg/creatinine ratio. The Mg/creatinine ratio reveals to possible bias due to incomplete urine collection. Furthermore, a normal urinary Mg/creatinine ratio indicates normal urinary Mg excretion and adequate Mg intake [28,29]. Our finding of normal Mg/creatinine ratios implies that there was complete urine collection and adequate Mg intake for all subjects. Furthermore, the intake of 100 mg of Mg from each product revealed no significant differences among the test products with respect to AUC_0-10h_ for serum Mg. Thus, the different mineralization levels of the tested mineral waters did not affect Mg bioavailability.

Mg homeostasis is strictly regulated by urinary excretion and urinary retention [7]. Thus, subjects with low initial serum Mg levels, suggesting a severe tissue Mg deficiency, have remarkably elevated urinary Mg retention [20]. In this study, the fasting Mg levels of all participants were within the normal range. Furthermore, prior studies have suggested an age-related decline in the capacity of the intestine to absorb dietary Mg [30,31]. This suggestion was supported by an experimental study that assesses enteral Mg absorption from a magnesium-rich mineral water and revealed a significant inverse correlation between age and Mg absorption [21]. However, this potential effect could be excluded in the present study due to the homogenous nature of the study population (mean age 24.2 ± 3.2 years). Furthermore, correlation analyses of the AUC_0-10h_ for serum Mg as well as the 24 h urinary Mg excretion and the age have not shown significant correlations between the parameters.

Mg bioavailability may be affected by formulation-related influences. Studies comparing solutions with capsules or enteric coated tablet formulations have found comparable Mg absorption for these products. However, increases in serum Mg levels were delayed after the consumption of enteric coated tablets [17,32]. Moreover, the formulation of a Mg supplement can affect urinary Mg excretion. Siener et al. showed that urinary Mg excretion increased by 40% after the ingestion of an effervescent tablet but by only 20% after the intake of a capsule [33]. However, in the present study, there was no difference in Mg absorption between the Mg supplement and the other test products.

The main finding of this study relates to the effect of different types of mineralization of mineral waters on the bioavailability of Mg. Notably, MW 3 was rich in SO_4_^2-^ (1463 mg/l), which is thought to potentially increase urinary volume and therefore decrease Mg bioavailability [34,35]. To our knowledge, no study has investigated the potential influence of SO_4_^2-^ in food on the bioavailability of Mg in humans. Additionally, MW 3 was low in HCO_3_^−^ (403 mg/l) compared with MW 1 (2451 mg/l), MW 2 (1816 mg/l), and MW 4 (1519 mg/l). To date, no study has determined the effect of HCO_3_^−^ on Mg bioavailability. Mineral water with higher concentration of calcium causes a significant increase in the urinary magnesium concentration [36]. However, correlation analysis of the 24 h urinary Mg excretion and the calcium content of mineral water has not shown a significant correlation between the parameters. Likewise, the Mg bioavailability was not influenced by different calcium concentrations in the tested mineral waters. Mg bioavailabilities were comparable and did not significantly differ for the products tested in this study. Therefore, neither SO_4_^2-^ content nor the content of HCO_3_^−^ or calcium influenced the bioavailability of Mg.

The current study had certain limitations. Participants received only a single dose of Mg from each of the test products. However, the efficiency of Mg absorption and retention (and therefore Mg bioavailability) can be enhanced by equally distributing Mg intake into smaller quantities over the course of a day. This increase might be due to the absorption of low quantities of Mg via transient receptor potential ion channels. Accordingly, distribution of Mg ingestion over a day could prevent saturation of these channels. In addition, the large volume of water consumed with the single bolus of test products may have decreased the transit time of Mg in the intestine. Therefore, total Mg uptake could have been limited by reduced exposure time [19,22]. These effects should be investigated in greater depth and considered in future trials.

## Conclusion

The results of serum and urine analysis indicated that Mg bioavailability was comparable for mineral waters with different mineralization levels, bread, and a dietary supplement. Specifically, Mg bioavailability was not influenced by the presence of SO_4_^2-^, HCO_3_,^−^ or calcium. Thus, mineral water with higher concentrations of Mg constitutes a calorie-free Mg source that contributes to optimal Mg supply. Future studies should be conducted to examine typical consumption patterns for mineral water because multiple portions consumed throughout the day may increase Mg bioavailability. Additional, the results of forthcoming intervention studies should complement the present findings by establishing the effect of mineral water with high Mg concentrations on cardiovascular risk factors.
